# Perturbation Dynamics of the Rumen Microbiota in Response to Exogenous Butyrate

**DOI:** 10.1371/journal.pone.0029392

**Published:** 2012-01-12

**Authors:** Robert W. Li, Sitao Wu, Ransom L. Baldwin, Weizhong Li, Congjun Li

**Affiliations:** 1 United States Department of Agriculture- Agricultural Research Service, Bovine Functional Genomics Laboratory, Beltsville, Maryland, United States of America; 2 Center for Research in Biological Systems, University of California, San Diego, California, United States of America; University of California Merced, United States of America

## Abstract

The capacity of the rumen microbiota to produce volatile fatty acids (VFAs) has important implications in animal well-being and production. We investigated temporal changes of the rumen microbiota in response to butyrate infusion using pyrosequencing of the 16S rRNA gene. Twenty one phyla were identified in the rumen microbiota of dairy cows. The rumen microbiota harbored 54.5±6.1 genera (mean ± SD) and 127.3±4.4 operational taxonomic units (OTUs), respectively. However, the core microbiome comprised of 26 genera and 82 OTUs. Butyrate infusion altered molar percentages of 3 major VFAs. Butyrate perturbation had a profound impact on the rumen microbial composition. A 72 h-infusion led to a significant change in the numbers of sequence reads derived from 4 phyla, including 2 most abundant phyla, Bacteroidetes and Firmicutes. As many as 19 genera and 43 OTUs were significantly impacted by butyrate infusion. Elevated butyrate levels in the rumen seemingly had a stimulating effect on butyrate-producing bacteria populations. The resilience of the rumen microbial ecosystem was evident as the abundance of the microorganisms returned to their pre-disturbed status after infusion withdrawal. Our findings provide insight into perturbation dynamics of the rumen microbial ecosystem and should guide efforts in formulating optimal uses of probiotic bacteria treating human diseases.

## Introduction

Volatile fatty acids (VFAs or short-chain fatty acids), such as acetate, butyrate and propionate, are major fermentation products of microorganisms in the rumen and hindgut. VFAs contribute up to 70% of the total metabolizable energy supply in ruminants [1]. In addition to their energetic or nutritional roles, VFAs are able to regulate animal physiology, including cholesterol synthesis and insulin and glucagon secretion. For example, butyrate is a preferred energy source for ruminal epithelial cells [2]. Most importantly, it has a multitude of cellular regulatory effects, such as modulating cell differentiation and motility, inducing apoptosis, and inhibiting cell proliferation [3], [4]. Intraruminal infusion of VFAs, including butyrate, has been used to study the effect of nutrient supply on milk secretion [5] and nutrient partition, liver physiology, lipid metabolism, and rumen wall development, as well as ruminal pH maintenance [6]–[8]. A decreased ratio of glucogenic (propionate)/lipogenic (acetate and butyrate) in ruminal VFAs could decrease hepatic gluconeogenesis [9], leading to a reduction in milk fat secretion [10]. Steady-state ruminal butyrate concentration reflects a delicate balance between butyrate production by rumen microbes and its clearance, mainly via epithelial absorption. Many attempts have been made to identify butyrate-producing bacteria in the human gut and the rumen [11]–[13]. However, little is known about the ecological and physiological role of predominant butyrate-producing bacteria in the rumen microbial ecosystem. The community level response of rumen microorganisms to exogenous VFAs, such as butyrate, has yet to be understood. While biochemical processes leading to butyrate biosynthesis is well described [14], the rumen microorganisms involved in this process have yet to be fully identified. In this study, we attempted to understand the effect of butyrate perturbation on the rumen microbial community composition and dynamics.

## Results

### Ruminal VFA concentrations

**Figure 1:**
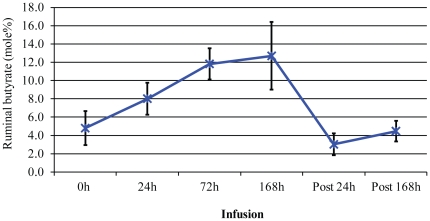
Butyrate molar percentages in the rumen of dairy cows in response to ruminal butyrate infusion. Error bars = SD. Post24 h and Post168 h = 24 h and 168 h after infusion withdrawal, respectively.

Ruminal sodium butyrate infusion rapidly affected rumen VFA concentration as well as VFA molar percentages. In the normal rumen of dairy cows (prior to the infusion), concentrations of three major VFAs, acetate, butyrate and propionate were 377.1, 19.5 and 23.4 mM, respectively. While the total VFA concentration, which is strongly diet- and feeding time-dependent, seemed high, their molar ratio (40∶2∶2) was within the normal range observed in the rumen [1]. 24 h after butyrate infusion ruminal butyrate concentration increased from 19.5 mM at the baseline to 23.7 mM (Table 1). This increase was associated with simultaneous reduction in the concentration of both acetate and propionate. After the initial decrease, the concentration of acetate in the rumen remained at the same repressed level as the infusion continued. However, butyrate concentration continued to increase with infusion and peaked at 168 h infusion, while propionate concentrations continued to decrease and reached its lowest level at 168 h. Within 24 h following withdrawal of butyrate infusion, ruminal butyrate concentration experienced a drastic reduction from the baseline level (43.1% reduction down to 11.1 mM). Concomitantly, the concentration of both acetate and propionate increased from their respective repressed levels. By 168 h post infusion (post168 h), the ruminal concentration of all 3 VFAs returned to the baseline. The ruminal molar proportion (percentage) of the 3 VFAs displayed a similar trend. At the baseline, acetate accounted for 89.5% of all ruminal VFAs while butyrate and propionate accounted for 4.8% and 5.7%, respectively. A 24 h infusion resulted in a significant increase in the ruminal butyrate molar percentage from 4.8% to 8.0% (*P* = 0.012; Figure 1). As the infusion progressed, the butyrate concentration continued to increase and reached a peak around 168 h post infusion (12.7%; *P* = 0.031). By 24 h after infusion withdrawal, a 76% decrease in the butyrate molar percentage was observed. This sudden reduction was temporary, and butyrate molar percentage also returned to its pre-infusion baseline at 168 h post infusion. Of note, temporal changes in the butyrate concentration as well as butyrate molar percentage composition in response to exogenous butyrate among individual dairy cows were not homogeneous. The ruminal butyrate percentage as well as the concentration reached its peak value at 72 h infusion in 2 of the 4 dairy cows studied. However, the both values did not reach their peak until 168 h infusion in the remaining 2 cows. The ruminal butyrate infusion using a buffered solution (pH 7.0) led to a slight increase in the rumen pH, however, the difference was insignificant (Table 1).

**Table 1:** The ruminal pH and volatile fatty acid concentrations (mM) in response to butyrate infusion.

	0 h	24 h	72 h	168 h	post24 h	post168 h
Acetate	377.1±56.3	247.0±13.9*	232.1±35.1*	239.6±35.9*	348.5±103.8	286.9±21.5*
Butyrate	19.5±5.4	23.7±7.1	32.5±4.0*	38.5±18.8*	11.1±3.8	14.5±4.0
Propionate	23.4±3.2	20.7±6.2	13.7±6.1*	12.5±6.4	20.5±3.8	21.1±4.5
pH	6.70±0.39	6.95±0.43	7.36±0.44	7.11±0.71	7.03±0.29	6.60±0.48

The numbers denote mean ± SD (*N* = 4).

*P<0.05 based on paired *t*-test. The pairs are pre- and post-infusion values of the same cow.

### The rumen microbiota composition

A total of 21 prokaryotic phyla were identified from the 16S rRNA gene sequences by RDP Classifier at a 95% confidence threshold. In the rumen microbial community of dairy cows at their mid-lactation, Bacteroidetes was predominant, represented by 70.87% of the 16S sequences in the undisrupted rumen microbial community. Firmicutes was the second most abundant phylum (22.20%), followed by Proteobacteria (3.25%), Fibrobacteres (1.52%), and Spirochaetes (1.11%). These 5 most abundant phyla accounted for ∼99% of the 16S sequences. A total of 10 phyla were identified in the rumen of all cows and in all time points tested. These phyla included Actinobacteria, SR1, Synergistetes, Tenericutes, and Verrucomicrobia, in addition to the 5 most abundant phyla. In addition, the phyla such as Cyanobacteria, Euryarchaeota, Planctomycetes, and TM7 were fairly common in the rumen communities. Additionally, archaea (Euryarchaeota) were widespread in the microbial communities of dairy cows even though our primers were not specifically designed to efficiently amplify the 16S rRNA gene sequences of archaeal origin.

The mean number of genera present in the rumen community of individual animals was 54.5 (ranging from 45 to 66). 26 genera were common to all 4 cows at all time points tested, probably representing a core microbiome of the bovine rumen community fed a silage-based diet. Collectively, 454 pyrosequencing allowed us to identify 137 prokaryotic genera in the rumen microbiota of dairy cows. *Prevotella* was the most abundant genus in the rumen community, represented by 64.82% of the 16S rRNA gene sequences in the rumen prior to the perturbation. Other most abundant genera included *Succiniclasticum* (12.09%), *Fibrobacter* (4.56%), *Ruminococcus* (2.90%), and *Treponema* (2.19%). There were a total of 9 genera with relative abundance greater than 1% in the community, such as *Butyrivibrio*, *Selenomonas*, *Moryella*, and *Succinivibrio*, in addition to the 5 most abundant genera described above. The rumen microbial community was dominated by the 10 most abundant genera, which accounted for 92.52% of the 16S rRNA gene sequences.

The microbial diversity and OTU-level composition were analyzed using CD-HIT-OTU, a novel clustering algorithm for rapid and accurate identification of microbial composition and diversity (http://weizhong-lab.ucsd.edu/cd-hit-otu/). On average, each rumen microbial community consisted of 127.25±4.42 OTUs (Operational Taxonomic Unit), ranging from 118 to 135. Colfolectively, 142 OTUs were identified in this study. Eighty two OTUs were present in the rumen microbial communities of all 4 cows in all time points tested and represented the core microbiome of the rumen microbial community. The lineage of the 4 most abundant OTUs, consisting of 57.07% of all 16S rRNA gene sequences in the rumen community prior to butyrate perturbation, can be traced back to the genus *Prevotella*. The 5^th^ most abundant OTU (OTU#5) was related to the genus *Moryella* (4.33%). The rumen microbial community was dominated by 20 OTUs; and these OTUs accounted to 89.39% of the 16S rRNA gene sequences.

### Dynamics of the rumen microbiota in response to butyrate infusion

Ruminal butyrate perturbation resulted in a profound change in rumen microbial composition at phylum-, genus-, and OTU- levels. Butyrate infusion led to a significant reduction in the most abundant phylum, Bacteroidetes. A 168 h ruminal butyrate infusion resulted in a reduction in its percentage composition from 70.87% prior to the infusion to 60.42%. The effect appeared instantaneous and long-lasting. The reduction was observed as early as 24 h after the perturbation was applied (reduction from 70.87% to 68.69%, *P*<0.05). However, a further reduction to 52.99% continued until 24 h post infusion. On the other hand, exogenous butyrate resulted in a significant increase in the relative abundance of Firmicutes, the second most abundant phylum. Ruminal butyrate infusion led to a significant increase in its relative abundance from 22.20% at 0 h to 29.61% (*P*<0.05) at 168 h. The increase continued and reached a plateau (35.86%; *P*<0.05) at 24 h post infusion. The relative abundance of as many as 6 phyla, including Bacteroidetes, Firmicutes, Fibrobacteres, Synergistetes, Planctomycetes, and Verrucomicrobia, was significantly impacted by exogenous butyrate at one or more time points.

**Figure 2:**
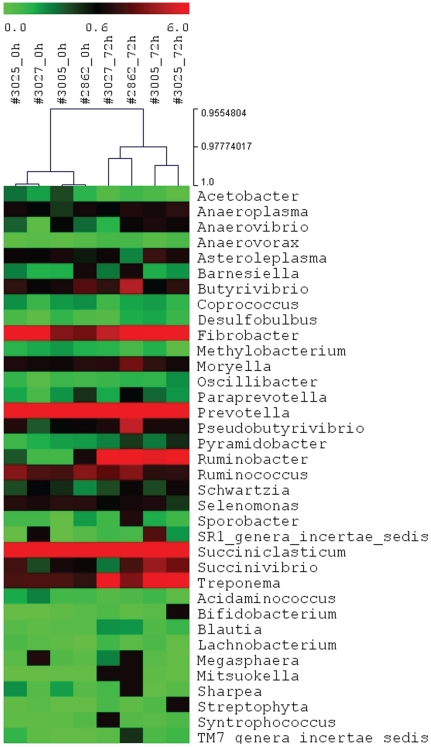
A heat-map of the rumen microbiota composition at a genus level. The 26 genera that shared by all samples tested (core microbiome) were displayed (*N* = 4). The scale was the percentage composition (log 2) based on the 16S sequences analyzed using RDP Classifier. A 72 h butyrate infusion induced a profound change in the rumen microbial composition, which allowed all 4 samples from this time point to be clustered using a Hierarchical Competitive Learning algorithm (HCL).

A total of 19 genera were significantly impacted by ruminal butyrate infusion (Table 2) at one or more time points. *Succinivibrio* was the only genus significantly affected as early as 24 h infusion. A 72 h-infusion led to a significant change in the relative abundance of 9 genera, displaying a clear separation between the microbiota of the two groups (0 h and 72 h-fusion) (Figure 2). The maximal impact of exogenous butyrate on the rumen microbial composition was readily observable at 168 h infusion and as many as 10 genera (18% of all genera in a given rumen microbial community) were affected.

**Table 2:** Ruminal butyrate infusion resulted in a profound change in genus-level microbial composition (mean percentage ± SD; *N* = 4).

Genus	0 h	24 h	72 h	168 h	post 24 h	post 168 h
*Acetobacter*	0.29±0.12	0.19±0.17	0.07±0.04	0.17±0.17	0.18±0.12	0.03±0.01*
*Acidaminococcus*	0.18±0.09	0.18±0.12	0.07±0.04	0.01±0.02*	0.02±0.01*	0.30±0.23
*Anaerovorax*	0.04±0.01	0.04±0.03	0.08±0.03*	0.12±0.07	0.11±0.07	0.05±0.03
*Asteroleplasma*	0.81±0.25	0.96±0.51	1.08±0.71	0.87±0.93	0.20±0.10*	1.37±0.66
*Blautia*	0.04±0.01	0.08±0.04	0.18±0.09	0.14±0.09	0.12±0.02*	0.10±0.09
*Coprococcus*	0.23±0.06	0.21±0.14	0.18±0.05	0.15±0.05*	0.24±0.21	0.20±0.16
*Desulfobulbus*	0.09±0.05	0.10±0.04	0.15±0.07	0.24±0.08*	0.18±0.11	0.13±0.06
*Fusobacterium*	0.00±0.01	0.02±0.01	0.08±0.05*	0.32±0.29	0.27±0.26	0.01±0.01
*Holdemania*	0.00±0.00	0.00±0.00	0.01±0.01	0.02±0.00*	0.02±0.02	0.00±0.01
*Methylobacterium*	0.20±0.03	0.13±0.07	0.12±0.07	0.12±0.03*	0.15±0.06	0.21±0.05
*Paraprevotella*	0.26±0.17	0.24±0.14	0.35±0.18*	0.26±0.21	0.25±0.17	0.29±0.32
*Prevotella*	64.82±2.61	61.52±4.54	48.45±4.77**	51.56±12.19	43.28±7.90*	59.69±8.61
*Ruminobacter*	0.43±0.47	1.49±1.41	8.25±3.05*	7.30±3.29*	9.76±3.41*	1.70±2.61
*Ruminococcus*	2.90±0.73	3.56±1.00	2.33±0.90	1.70±0.41*	2.13±0.50	3.14±0.77
*Selenomonas*	1.39±0.20	1.30±0.73	0.85±0.34*	0.63±0.11*	0.59±0.23*	1.29±0.40
*Streptophyta*	0.03±0.01	0.05±0.03	0.04±0.04	0.05±0.05	0.08±0.07	0.07±0.03*
*Succinimonas*	0.03±0.03	0.08±0.12	0.16±0.21	0.66±0.30*	1.40±1.51	0.07±0.10
*Succinivibrio*	1.11±0.73	1.95±1.10*	2.37±1.52	1.77±0.87	2.27±2.48	3.24±2.76
*Treponema*	2.19±0.29	3.61±1.38	5.91±2.05*	5.97±1.95*	4.04±1.73	2.57±0.90

*P<0.05;

**P<0.001.

**Figure 3:**
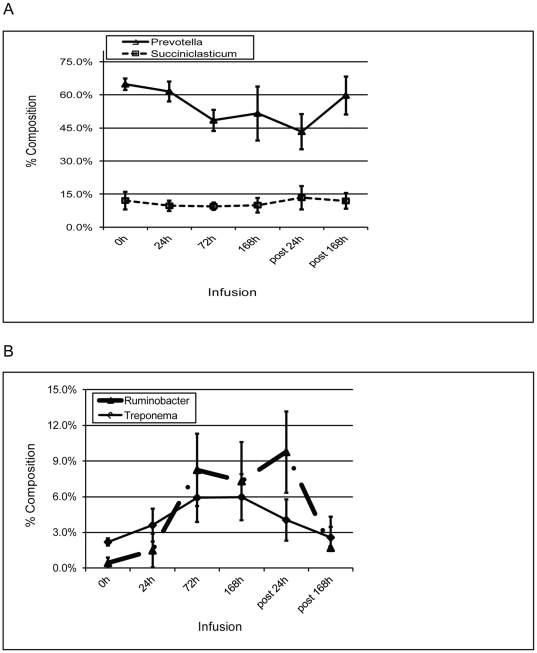
Temporal changes in the relative abundance (% reads) of A) 2 most dominated genera, *Prevotella* (solid line) and *Succininclasticum* (dashed line), and B) *Ruminobacter* and *Treponema*, in the rumen microbial community of dairy cows in response to butyrate infusion. Error bars represent SD of 4 samples. Normalized percentages represent the number of 16S rRNA gene sequences assigned to a given genus.

Exogenous butyrate resulted in a drastic reduction in the most abundant genus, *Prevotella* (Figure 3A). *Prevotella* consists of a group of bacteria with predominant roles in protein metabolism. Its relative abundance in the control rumen microbiota (0 h) was at 64.82%. A 72-h butyrate infusion reduced its abundance to 48.45%. However, the relative abundance of the second most abundant genus, *Succiniclasticum*, was little changed (Figure 3A). The differences in microbiota composition induced by the infusion appeared to be genus-specific and did not seem to be synchronized. As Figure 3B shows, the relative abundance of *Treponema*, the 5th most abundant genus, reached its plateau between 72 h and 168 h. On the other hand, the relative abundance of *Ruminobacter* was significantly increased starting at 72 h (Figure 3B).

**Figure 4:**
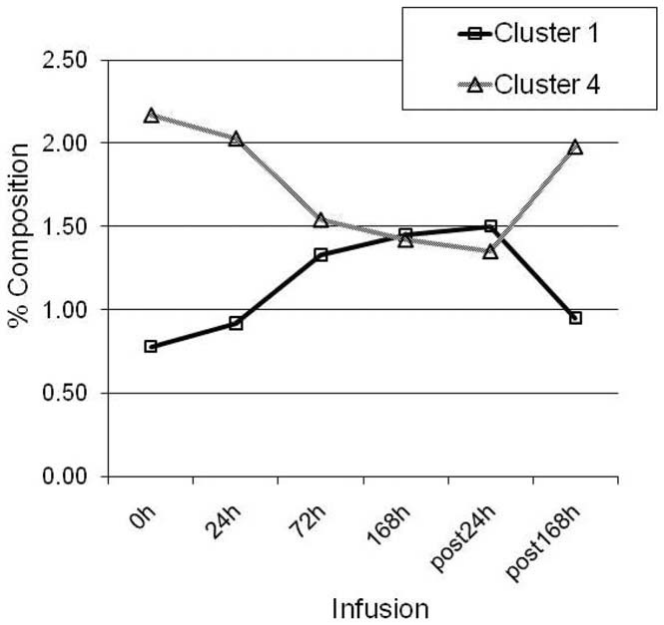
Temporal profiles of the relative abundance (% reads) of Operational Taxonomical Units (OTUs) of Clusters #1 and 4. Clusters were generated based on Self Organizing Maps (SOM). Percentage composition represents mean normalized percentages of OTUs of 4 samples (*N* = 4).

**Figure 5:**
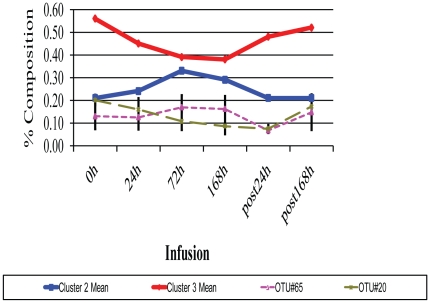
Temporal profiles of the relative abundance (% reads) of Operational Taxonomical Units (OTUs) of Clusters #2 and 3. Clusters were generated based on Self Organizing Maps (SOM). Group Mean represents mean normalized percentages of OTUs of 4 samples in this cluster (*N* = 4). A temporal profile of representative OTUs (OTU#65 and OTU#20) that belonged to clusters#2 and 3, respectively was shown as a reference. Error bars represent SD of 4 samples.

**Figure 6:**
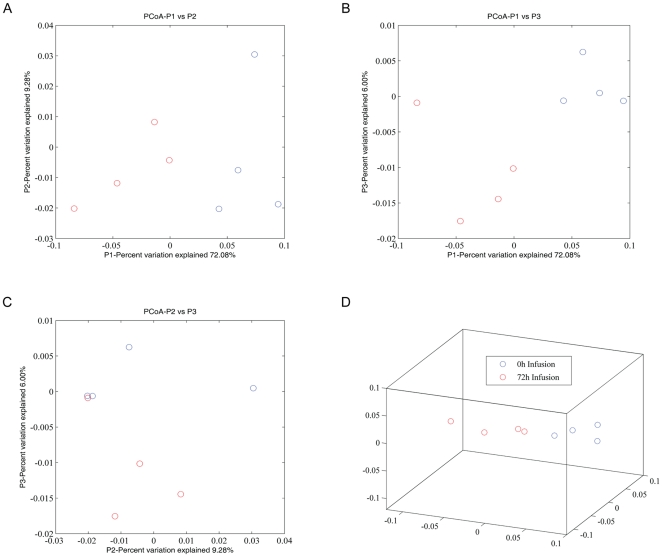
Principal Coordinates Analysis of weighted UniFrac values between pre-infusion (0 h; blue) and 72 h infusion (red) (*N* = 4). (A) Visualization of the first two dimensions (un-scaled); (B) Visualization of the first and third dimensions (un-scaled); (C) Visualization of the second and third dimensions (un-scaled); (D) in 3-D.

43 OTUs were significantly impacted by infusion at a stringent cutoff FDR<5%, representing ∼52% of the core rumen microbiome (Table 3). A Self Organizing Map (SOM) analysis, using an unsupervised learning technique to generate a type of artificial neural networks, allowed us to cluster all 82 OTUs that formed the rumen core microbiome into 4 distinct clusters. Clusters1 and 4 were the largest and contained 35 and 30 OTUs, respectively (Figure 4). These 2 clusters displayed an opposite trend in their temporal patterns in response to butyrate infusion. The relative abundance of OTUs in Cluster 1 tended to increase as infusion progressed and did not reach their peak until the 24 h post infusion (post24 h). On the other hand, the relative abundance of OTUs in Cluster 4 started to a descending slope as the infusion began; and the lowest level of the abundance was observed only at post24 h (Figure 4). OTUs in Cluster 1 were phylogenetically diverse, involving in at least 6 phyla/genera, ranging from *Moryella*, *Ruminococcus*, *Ruminobacter*, *Treponema*, to *Clostridium* and *Selenomonas*. However, the majority of OTUs belonging to *Prevotella* were concentrated in Cluster 4. OTUs in Cluster 2 increased in their relative abundance and reached their plateau at 72 h infusion and then started to decrease (Figure 5). OTU#65, annotated to *Selenomonas ruminantium*, was a typical representative of this cluster. Cluster 3, on the other hand, started its descending slope in its relative abundance and reached its lowest point at 168 h infusion (Figure 5), which was different from Cluster #4, where the lowest abundance level was not reached until 24 h post infusion (Figure 4). OTUs annotated to the genus *Prevotella* were distributed in 3 clusters, Clusters#2, #3, and #4, suggesting its functional divergence. 72 h butyrate infusion induced a sufficient change in the ruminal microbial composition at both genus and OTU levels, which allowed 4 samples at this time point to be clustered using HCL (Figure 2) and PCoA analysis by Fast UniFrac (Figure 6). Infusion did not seem to affect biodiversity indices, such as ACE and Chao1, in the rumen. Of interest, infusion may have created a favorable rumen environment that allowed more microbial genera to be established as the number of genera was higher at 168 h infusion (60.3±4.9) than at the baseline (52.8±3.3), coinciding with the maximal butyrate molar percentage in the rumen (Figure 1). However, it seemed paradoxical that the number of OTUs in this time point was lower than the baseline (124.5±3.5 vs 130.5±2.4). Nevertheless, understanding temporal changes in the rumen microbiota composition in response to butyrate infusion will undoubtedly facilitate rapid identification and isolation of novel butyrate-producing bacteria and provide insight into their contributions in the rumen microbial ecosystem output.

**Table 3:** Ruminal butyrate infusion resulted in a profound change in the species-level rumen microbial composition (mean percentage ± SD; *N* = 4).

Name	0 h	24 h	72 h	168 h	post24 h	post168 h	Annotation
OTU1	20.31±1.00	18.49±2.01	13.67±1.53**	13.81±4.69	13.56±3.81*	17.19±2.50	g:Prevotella
OTU2	15.37±1.74	15.27±2.43	13.81±4.63	10.45±2.55*	9.32±3.10*	14.98±5.03	g:Prevotella
OTU3	13.20±0.76	10.96±1.00	6.96±0.98**	8.24±2.83*	8.19±3.02*	10.23±2.96	g:Prevotella
OTU6	8.19±1.39	7.39±0.95	5.41±1.19	5.19±1.53*	4.32±1.63*	7.42±0.96	s:P.ruminicola
OTU5	4.33±0.80	4.50±0.89	5.57±0.80	6.22±0.95*	6.52±1.09*	5.86±1.30	g:Moryella
OTU11	3.04±0.86	3.55±0.96	4.14±2.01	5.01±1.09	5.83±1.77*	3.25±2.47	s:R. flavefaciens
OTU7	2.69±0.61	3.51±0.92	5.38±0.66**	5.00±1.42*	4.85±0.93*	3.51±2.39	s:R.flavefaciens
OTU10	2.62±0.60	2.98±0.61	3.77±1.58	4.09±0.72*	5.58±1.51*	2.82±1.95	f:Ruminococcaceae
OTU16	2.47±0.45	2.35±0.64	2.50±0.86	3.27±0.65	3.49±0.38*	3.07±0.80	f:Lachnospiraceae
OTU12	1.50±0.39	2.13±0.58	3.10±0.39*	3.09±1.30	2.53±0.78	2.03±0.70	o:Bacteroidales
OTU9	0.54±0.41	0.94±0.60	2.43±0.48**	3.07±1.43*	4.84±3.26*	1.32±1.70	o:Aeromonadales
OTU52	0.40±0.04	0.63±0.19	0.86±0.34	0.86±0.15**	0.63±0.22	0.47±0.11	g:Treponema
OTU77	0.37±0.08	0.51±0.10	0.95±0.33	0.77±0.15*	0.48±0.12	0.46±0.17	g:Treponema
OTU71	0.36±0.15	0.30±0.06	0.63±0.12	0.76±0.18*	0.65±0.32	0.24±0.15	g:Clostridium
OTU74	0.34±0.09	0.28±0.05	0.42±0.09	0.62±0.16*	0.64±0.27	0.22±0.08	g:Selenomonas
OTU14	0.30±0.10	0.30±0.10	0.15±0.03	0.10±0.05*	0.09±0.03*	0.30±0.14	g:Prevotella
OTU56	0.21±0.08	0.26±0.13	0.26±0.18	0.24±0.32	0.05±0.03*	0.33±0.17	g:Asteroleplasma
OTU53	0.20±0.13	0.50±0.32	1.45±0.43*	1.35±0.68*	1.81±0.96*	0.49±0.50	g:Ruminobacter
OTU20	0.20±0.02	0.16±0.05	0.11±0.04	0.09±0.04*	0.08±0.02**	0.17±0.05	g:Prevotella
OTU108	0.18±0.04	0.27±0.12	0.32±0.14	0.37±0.07*	0.28±0.09	0.20±0.05	g:Treponema
OTU133	0.15±0.03	0.21±0.10	0.16±0.07	0.19±0.13	0.05±0.03*	0.15±0.06	g:Anaeroplasma
OTU35	0.14±0.10	0.26±0.20	0.55±0.08**	0.68±0.30*	0.83±0.49*	0.30±0.39	g:Ruminobacter
OTU19	0.11±0.03	0.11±0.02	0.14±0.08	0.08±0.05	0.05±0.03*	0.11±0.04	g:Prevotella
OTU76	0.09±0.01	0.10±0.04	0.09±0.04	0.26±0.09*	0.31±0.34	0.13±0.06	o:Bacteroidales
OTU126	0.09±0.02	0.05±0.02	0.06±0.02	0.05±0.01*	0.05±0.01*	0.08±0.02	g:Prevotella
OTU26	0.08±0.02	0.07±0.01	0.05±0.01	0.08±0.03	0.05±0.02*	0.05±0.01	g:Prevotella
OTU34	0.08±0.02	0.07±0.02	0.04±0.02	0.04±0.01*	0.04±0.02*	0.08±0.03	g:Prevotella
OTU50	0.06±0.03	0.04±0.02	0.01±0.01	0.00±0.00*	0.00±0.00*	0.02±0.03	g:Prevotella
OTU49	0.05±0.02	0.04±0.01	0.01±0.01	0.00±0.01*	0.00±0.00*	0.03±0.01	g:Prevotella
OTU22	0.04±0.01	0.06±0.02	0.09±0.02	0.07±0.04	0.12±0.07*	0.06±0.05	o:Clostridiales
OTU128	0.03±0.05	0.04±0.04	0.24±0.15	0.35±0.21*	0.19±0.29	0.01±0.01	c:Alphaproteobacteria
OTU67	0.03±0.01	0.02±0.02	0.01±0.01	0.01±0.00*	0.00±0.00**	0.02±0.02	g:Prevotella
OTU141	0.02±0.01	0.02±0.01	0.04±0.02	0.04±0.01*	0.01±0.00	0.02±0.01	g:RFN20
OTU139	0.02±0.02	0.02±0.01	0.07±0.01	0.07±0.02*	0.06±0.03	0.04±0.04	o:Clostridiales
OTU79	0.02±0.01	0.02±0.00	0.01±0.00	0.01±0.01	0.00±0.00*	0.02±0.02	g:Prevotella
OTU58	0.02±0.00	0.02±0.01	0.03±0.01	0.02±0.01	0.01±0.00*	0.02±0.01	g:Paludibacter
OTU91	0.01±0.01	0.01±0.01	0.00±0.00	0.01±0.00	0.00±0.00*	0.01±0.00	g:Prevotella
OTU59	0.01±0.01	0.01±0.01	0.01±0.01	0.01±0.00	0.05±0.03*	0.01±0.01	g:Ruminococcus
OTU92	0.01±0.01	0.01±0.01	0.01±0.01	0.01±0.00	0.00±0.00*	0.01±0.01	g:Prevotella
OTU110	0.01±0.01	0.02±0.01	0.02±0.01	0.04±0.01*	0.03±0.01*	0.01±0.01	g:Treponema
OTU107	0.01±0.00	0.01±0.00	0.01±0.00	0.01±0.01	0.02±0.01*	0.01±0.00	o:Clostridiales
OTU113	0.01±0.01	0.01±0.01	0.02±0.02	0.04±0.03	0.04±0.02*	0.02±0.02	g:Coprococcus
OTU131	0.00±0.01	0.01±0.01	0.01±0.01	0.02±0.02	0.02±0.01*	0.00±0.00	o:Aeromonadales

**FDR<5% and *P*<0.001.

*FDR<5% and *P*<0.05.

The consensus sequence of each OTU was annotated to the closest lineage using FR-HIT against the GreenGene database.

s: = species; g: = genus; f: = family; o: = order; and c: = class.

A stringent cutoff at a global false discovery rate or FDR<5% was used. OTUs were sorted based on their relative abundance in the rumen microbial community prior to butyrate infusion (0 h) in a descending order.

## Discussion

The rumen microbiota plays an essential role in nutrient production and utilization in ruminants [15], [16]. Efficient microbial transformation of plant fibers results in production of VFAs, which are subsequently used to produce meat and milk for human consumption. Rumen fermentation is poorly understood process controlled by the interacting rumen microbiota constituents. Understanding of microbial interactions and dynamics in the rumen microbial ecosystem should provide a scientific basis for successful manipulation of ruminal fermentation for optimal outcomes. In this study, we characterized temporal changes of the rumen microbiota of dairy cows in their mid-lactation in response to an exogenous butyrate disturbance. We identified 21 prokaryotic phyla in the rumen microbial community, which were dominated by 2 phyla, Bacteroidetes and Firmicutes. Together, these 2 phyla accounted for up to 93% of all 16S rRNA gene sequences. Compared to the rumen of 12-month-old steers fed a similar hay-based diet, where 16S rRNA gene sequences can be assigned to 15 bacterial phyla [16], the rumen microbiota of mid-lactating dairy cows displayed some unique features. One of the salient features was prominent establishment of bacteria from a candidate phylum SR1 in the community, which was absent from the rumen of 12-month-old steers. The role of these bacteria in the rumen microbial ecosystem remains unknown.

Butyrate is a major microbial fermentation product in the forestomach (rumen) of ruminants and the lower digestive tract of humans and all animal species and contributes to approximately 70% of the daily metabolizable energy requirement of ruminants and ∼10% for humans [1]. In addition to serve as a preferred energy source for the rumen epithelia and human colonocytes, butyrate is an important regulator of host physiology and acts as a signaling molecule in epithelial cells [3], [17]. Butyrate has important implications in human health as well, especially in gastrointestinal disorders (colon cancer and enterocolitis) and cardiovascular diseases, including its role in development of normal colon mucosa and the prevention of colon cancer [18]. Additionally, butyrate irrigation has been used to treat colitis. Butyrate production in the gastrointestinal (GI) tract, which has recently been shown to play a special role in modulating bacterial energy metabolism in the gut ecosystem [19], depends on diets, microbial species and their relative abundance present in the gut ecosystem, and gut transit time. Over the years, extensive searches have been conducted to identify butyrate-producing bacteria in the rumen of both domestic and wild ruminants and the hindgut of humans [11], [20]. In the human hindgut, butyrate-producing bacteria are mainly from the phylum Firmicutes (the clostridial clusters IV and XIVa; [21]) and 80% of butyrate-producing isolates belong to the XIVa cluster of Gram^+^ bacteria, such as species from *Butyrivibrio*, *Eubacterium* and *Roseburia* [11]. In addition, cluster IV includes several species that are butyrate-producing, such as *Faecalibacterium prausnitzii*, *Subdoligranulum variabile*, and *Anaerotruncus colihominis* [20]. In the rumen, butyrate-producing bacteria as a functional group likely had a distinct phylogenetic profile. *Butyrivibrio fibrisolvensis* is a major butyrate producing bacterium and responsible for fiber digestion and utilization in the rumen [22]. Our results show that *Butyrivibrio* was among the 5 most abundant genera and accounted for 1.46% of all 16S sequences in the undisturbed rumen ecosystem. The major butyrate-producing bacteria in the colon of monogastric species including humans, such as *Eubacterium*, *Anaerostipes*, *Roseburia*, and *Faecalibacterium*, were very rare in the rumen. *Butyrivibrio* (1.46%) and *Pseudobutyrivibrio* (0.82%) were probably predominant butyrate producers in the rumen microbial ecosystem. Interestingly, exogenous butyrate seemingly had a stimulating effect on the native butyrate-producing bacterial population. A 168-h infusion resulted in an increase in the relative abundance of *Butyrivibrio* and *Pseudobutyrivibrio* by 65% and 141%, respectively, albeit not statistically significant, compared to their pre-infusion levels. Other butyrate-producing bacteria, such as *Roseburia*, also responded favorably to exogenous butyrate. If these patterns were confirmed, it is likely to isolate many previously unrecognized butyrate-producing bacteria from the rumen, especially from those belonging to Clusters#1 and 2 (Figures 4 and 5). The expansion in the relative abundance of these butyrate-producing bacteria may at least partially account for the observed expansion of the phylum Firmicutes in response to butyrate infusion.

Ruminal butyrate infusion resulted in a doubling in butyrate concentration from 19.5±5.4 mM at the basal level to 38.5±18.8 mM at 168 h infusion at the expense of both ruminal acetate and propionate concentrations (Table 1). As a result, butyrate molar percentage increased significantly from 4.8% in the undisturbed rumen (the baseline) to 12.7% at 168 h infusion (*P*<0.05). These drastic changes had a profound impact on ruminal microbial composition, community structures, and metabolic potentials because the substrate-product equilibrium in the rumen microbial ecosystem was altered. At 168 h infusion, the relative abundance of 10 genera was significantly changed (*P*<0.05), coinciding with the maximal change in both ruminal butyrate concentration and molar percentage ratios of 3 major VFAs. The perturbation also led to changes in relative abundance of as many as 26 OTUs representing 32% of OTUs in the core microbiome. A sudden withdrawal of exogenous butyrate had a repressive effect on ruminal butyrate biosynthesis and led a 43% reduction in ruminal butyrate concentration from the basal level (19.5 mM) to 11.1 mM at 24 h post infusion. Concurrent with this reduction was the changes in relative abundance of the maximal number of OTUs observed (33) at FDR<5% at 24 h post infusion, suggesting adaptation of the rumen ecosystem to substrate availability may be time-dependent. However, both ruminal butyrate concentration and butyrate molar percentage returned to the basal level at 168 h post infusion. By 168 h post infusion, the genus-level rumen microbial composition showed a high-degree of similarity (>81%) to their normal (undisturbed) status based on Bray-Curtis similarity coefficients (Table 4) while the normal (0 h) and 168 h infusion shared the least similarity at the genus level. Cluster analysis indicates that the genus-level composition of the rumen microbial communities was indistinguishable between 0 h (normal/undisturbed) and post168 h infection (data not shown). These data suggest that the rumen microbial ecosystem displayed substantial resilience to the short-term change in butyrate concentration.

**Table 4:** Bray-Curtis similarity matrix.

	0 h_#1	0 h_#2	0 h_#3	0 h_#4	168 h_#1	168 h_#2	168 h_#3	168 h_#4	p168 h_#1	p168 h_#2	p168 h_#3	p168 h_#4
0 h_#1												
0 h_#2	86.79											
0 h_#3	83.90	84.23										
0 h_#4	80.31	82.07	81.78									
168 h_#1	74.04	69.04	69.75	64.04								
168 h_#2	76.65	73.41	72.66	68.29	81.19							
168 h_#3	78.29	80.34	78.28	74.07	69.36	74.19						
168 h_#4	75.23	73.64	73.01	69.85	80.70	83.82	72.61					
p168 h_#1	86.21	81.48	79.71	75.84	77.22	73.46	72.04	75.79				
p168 h_#2	82.27	85.58	86.20	82.79	69.39	72.31	80.17	74.97	79.73			
p168 h_#3	83.25	80.68	82.77	75.18	77.25	79.18	78.47	76.89	79.73	80.30		
p168 h_#4	77.12	82.34	79.71	83.59	63.91	66.89	77.95	69.03	74.02	85.71	77.10	

The relative abundance was first square root transformed.

Bray-Curtis similarity coefficients were calculated using PRIMER v6. p168 h = post168 h.

Butyrate plays an important role in host physiology and gut health. A persistent elevated level of butyrate in the GI tract could have positive impacts on nutrient utilization efficiency in ruminants and the prevention and treatment of colon diseases in nonruminants. It has been known that the metabolic outputs of the gut microbial ecosystem depend on both diets and gut environmental factors, such as pH [12], [21]. Factors affecting gut production of butyrate have been investigated. Higher-fiber intake in cattle tends to increase the population of major butyrate-producing bacteria, *Butyrivibrio* [22], resulting in an increase of ruminal butyrate concentration, whereas high-energy feeds lead to the suppression of these bacteria. Resistant starch is known to be butyrogenic in humans [23] and modify the composition of the gut microbiota and elevate butyrate production in human flora-associated rats [24]. In healthy obese human subjects, lower carbohydrate intakes lead to a significant reduction in fecal butyrate with a proportional decrease in the abundance of the *Eubacterium rectale/Roseburia* subgroup of the clostridial cluster XIVa [25]. Formulating a diet aiming at a selective stimulation of butyrate-producing bacteria as well as non-butyrate producing bacteria, which may indirectly affect butyrate production via metabolic cross-feeding [26], can have a desired effect on butyrate biosynthesis. The key to a successful dietary regime is to use metagenomic tools to holistically monitor dietary impacts on the microbial composition and prevent overexpansion of lactic acid producing bacteria, which may contribute to rumen acidosis in cattle.

Introducing butyrate-producing bacteria into the gut ecosystem has been suggested as a means to treat and prevent colon cancer and enterocolitis, including inflammatory bowel diseases. Indeed, an oral administration of *Butyrivibrio fibrisolvens*, a species isolated from the bovine rumen with a high capacity to produce butyrate, in mice results in a significant increase in the rate of fecal butyrate production and decreases the formation of experimentally induced aberrant crypt foci [27] and alleviated the symptoms of experimentally induced enterocolitis [28]. An ideal probiotic should possess properties able not only to colonize in the desired gut location for a prolonged period of time but also to maintain its high production potentials. Previous successes in establishing introduced bacteria into the rumen have involved species with unique metabolic capabilities that can fill empty habitat niches [29]. Butyrate producing bacteria are phylogenetically diverse [11], [12], [20] and host species may display their unique microbial profiles of butyrate biosynthesis. In addition, the tendency to resist colonization of foreign probiotic bacteria by the indigenous microflora and apparent host specificity of the gut bacterial community [29] require us to have a holistic understanding of dynamics of the gut microbial ecosystem in response to perturbation before a successful probiotic strategy can be optimized.

## Materials and Methods

### Cows and diet

Four ruminally-cannulated Holstein cows in mid-lactation were used in this study. These multiparous cows fed *ad libitum* standard lactation rations as a Total Mixed Ration (TMR; 50% corn silage and 50% concentrate at a dry matter basis). Diets were formulated to provide or exceed NRC dietary recommendations for lactation. Complete dietary chemical composition was determined on daily samples of TMR composited weekly. All animal procedures were conducted under the approval of the Beltsville Area Institutional Animal Care and Use Committee and the University of Maryland Animal Care and Use Committee. Cows were moved to a tie stall barn for adaptation and acclimation at least five days prior to the infusion experiment. Rumen contents (solids and rumen juice) of the normal dairy cows (just prior to infusion or 0 h) were collected from different locations inside the rumen. Infusion of butyrate was initiated immediately following 0 h sampling and thereafter continued for 168 h (7 days) at a rate of 5.0 L/d of a 2.5 M solution such that 12.5 moles were infused daily in a buffered saliva solution (pH 7.0; 3.8% KHCO_3_, 7.3%NaHCO_3_) as a continuous infusion. This amount of butyrate was selected to represent greater than 105% of daily anticipated metabolizable energy (ME) intake and ranged from 15.5 to 25.7. After 168 h infusion, cows were returned to a standard ration without ruminal infusion for an additional 168 h. Rumen contents were serially collected at 0, 24, 72, and 168 h of infusion, and 24 and 168 h post infusion through rumen fistulas. The rumen liquor pH was measured using a standard pH meter. The liquid fraction of rumen contents passed through a 300-µM metal sieve was collected and centrifuged at 16,000× *g* for 3 min. The supernatant was decanted and the remaining solid materials were snap frozen in liquid nitrogen and stored in −80C until DNA extraction.

### Metagenomic DNA extraction, amplicon preparation and pyrosequencing

Metagenomic DNA extraction and sequencing were essentially same as described [16]. Briefly, a QIAamp DNA stool kit (Qiagen, Valenica, CA) with modifications was used for DNA extraction. A six-minute incubation at 95°C was used to replace the 70°C lysis recommended in the standard protocol. A 570-bp region of the 16S rRNA gene (*E. coli* position 357 to 926) containing hypervariable regions V3- V5, selected because of their high variability [30], of the 16S rRNA gene, was amplified from 40 ng of metagenomic DNA with 8-bp sample-specific barcoded primers using 2.5 units of AccuPrime Taq DNA Polymerase High Fidelity (Invitrogen, Carlsbad, CA) in a 50-µl reaction buffer containing 200 nM primers, 200 nM dNTP, 60 mM Tris-SO_4_, 18 mM (NH4)_2_SO_4_, 2.0 mM MgSO4, 1% glycerol, and 100 ng/ul bovine serum albumin (New England Biolabs, Ipswich, MA). PCR was performed using the following cycling profile: Initial denaturing at 95°C for 2 minutes followed by 25 cycles of 95°C 30 s, 50°C 30 s, and 72°C 120 s. The amplicons were generated from each metagenomic DNA sample separately, purified using a Agencourt AMPure XP kit (Beckman Coulter Genomics, Danvers, MA), and quantified using a QuantiFluor fluorometer (Promega, Madison, WI). The amplicons from individual samples were pooled in equal mass (molar) ratios. The amplicon pool at the desired size (∼672 bp including primers and adaptors) was excised from 1.0% agarose gel and purified using a QIAquick Gel Extraction Kit (Qiagen). The purified amplicon library was further verified and quantified using a BioAnalyzer 2000 (Agilent) and subject to 454/Roche pyrosequencing.

Unidirectional pyrosequencing of amplicon libraries was performed according to the manufacturer's instructions with a modification (App No 001-2009, Roche Applied Science, Indianapolis, IN). This modification, using a specific fusion primer design, accommodates amplification using the GS FLX Titanium emPCR Kits (Lib-L). Pyrosequencing was conducted using a GS FLX Titanium System (Roche) following the manufacturer's protocol. A total of 24 samples (4 cows at 6 time points, 0, 24, 72, and 168 h of infusion, and 24 and 168 h post infusion) were sequenced using 454 FLX Titanium pyrosequencing. The number of raw sequences reads for each sample was 30,606.58±5,261.7 (mean ± SD) at ∼450 bp/read (NCBI SRA accession# SRA043755.1).

### Sequence analysis

The raw reads were first decoded based on 8-bp bar codes; their quality was checked and artifacts were removed as previously described [16]. Sequences were filtered to remove low-quality reads and initially analyzed using BLAST against the GreenGene database. Sequence reads shorter than 200 bp were excluded and resultant quality sequence reads were then annotated using RDP Classifier [31] from the Ribosomal Database Project (release 10) at a 95% confidence threshold for their taxonomic classification and phylogenetic inference. Raw read counts were normalized. A square root transformation was applied to the relative abundance data. Bray-Curtis similarity matrix was then calculated using PRIMER v6 software.

The 16S rRNA gene sequences were also analyzed using CD-HIT-OTU [32] for phylogenetic inference at the OTU level. This algorithm uses a greedy incremental clustering process to identify OTUs from 16S rRNA gene tags, which involves 3 major steps: raw read filtering and trimming, selection of error-free reads, and clustering selected representative reads into individual OTUs at a user-specific cutoff (97% identity). The program avoids over estimation of OTUs, a common problem for many existing programs, and results in a rapid and more accurate estimation of microbial diversity in complex microbial ecosystems. OTUs identified using CD-HIT-OTU were then annotated using FR-HIT [33] against the GreenGene database. Statistical analysis was carried out according to MetaStats [34]. Self-organizing maps (SOMs) were generated according to unsupervised learning.

The 16S sequences were further analyzed using Fast UniFrac [35]. Briefly, the core set of the 16S GreenGene database was first downloaded, and the input 16S sequences were analyzed using MegaBLAST. The resultant hit table was input into the Fast UniFrac server for Principal Coordinates Analysis (PCoA).
